# Joint Bearing and Range Estimation of Multiple Objects from Time-Frequency Analysis

**DOI:** 10.3390/s18010291

**Published:** 2018-01-19

**Authors:** Jeng-Cheng Liu, Yuang-Tung Cheng, Hsien-Sen Hung

**Affiliations:** Department of Electrical Engineering, National Taiwan Ocean University, No.2 Pei-ning Rd., Keelung 20224, Taiwan; d96530001@mail.ntou.edu.tw (J.-C.L.); b0221@mail.ntou.edu.tw (H.-S.H.)

**Keywords:** DOA, HHT, ULA, MEMS, AUV, TFD

## Abstract

Direction-of-arrival (DOA) and range estimation is an important issue of sonar signal processing. In this paper, a novel approach using Hilbert-Huang transform (HHT) is proposed for joint bearing and range estimation of multiple targets based on a uniform linear array (ULA) of hydrophones. The structure of this ULA based on micro-electro-mechanical systems (MEMS) technology, and thus has attractive features of small size, high sensitivity and low cost, and is suitable for Autonomous Underwater Vehicle (AUV) operations. This proposed target localization method has the following advantages: only a single snapshot of data is needed and real-time processing is feasible. The proposed algorithm transforms a very complicated nonlinear estimation problem to a simple nearly linear one via time-frequency distribution (TFD) theory and is verified with HHT. Theoretical discussions of resolution issue are also provided to facilitate the design of a MEMS sensor with high sensitivity. Simulation results are shown to verify the effectiveness of the proposed method.

## 1. Introduction

With the rapid development of marine activities in recent years, underwater detection technologies have been applied extensively in areas such as offshore oil exploration, marine environmental pollution monitoring, crash vehicle search, aquaculture, marine scientific data collection, unmanned underwater vehicle, and marine fisheries. In a passive sonar system, an array of acoustic hydrophones is used as a sensing device to observe the sound wave generated by the underwater target itself, and the received signal is processed to decide the target’s direction-of-arrival (DOA) (bearing or azimuth) and range [1]. 

Traditional DOA estimation technology makes use of the time difference or phase difference for a sound wave reaching each element of the array to estimate the bearing of the target [2,3]. It is intended to separate the background noise or interference from the target source signal and fetching the meaningful target’s information. In general, the analysis method varies according to the stationary or nonstationary characteristics of the target signal. In the analysis of stationary sound signals, traditional popular methods include Fast Fourier transform (FFT) [4,5]. The nonstationary signal analysis methods include short-time Fourier transform [6], wavelet transform [7] and Wigner–Ville distribution (WVD) [8,9]. Recently, the Hilbert-Huang transform (HHT) [10] was proposed and found to be very effective for analyzing nonlinear and/or nonstationary signals in various areas, such as ocean wave characterization, etc. [11]. Other DOA estimation approaches include Multiple Signal Classification (MUSIC) [12,13,14] and Estimating of signal parameters via rotational invariance techniques (ESPRIT) [15]. The MUSIC and ESPRIT algorithms primarily use the eigen-decomposition of the auto-correlation matrix of the received signal to determine the DOAs of multiple targets. In both algorithms, the number of sensors must be greater than that of the targets. Moreover, multiple snapshots are required to avoid rank-deficient problems. For better estimation performance, the array aperture needs to be large, leading to difficulty of eigen-structure computation. 

Traditional sonar and hydrophone are very large in size. However, underwater vehicles, such as Autonomous Underwater Vehicles (AUV), are very compact, so that large-size sonar cannot be deployed from them. Therefore, a recent trend has focused on the development of small-size sensors with high sensitivity via micro-electro-mechanical systems (MEMS) technology [16]. In the past, a MEMS-based acoustic linear uniform array of hydrophones for underwater applications was proposed by our research team [17]. Motivated by our previous work, we developed a novel HHT-based method for joint bearing and range estimation of multiple targets in this paper. 

## 2. Data Model

**Figure 1:**
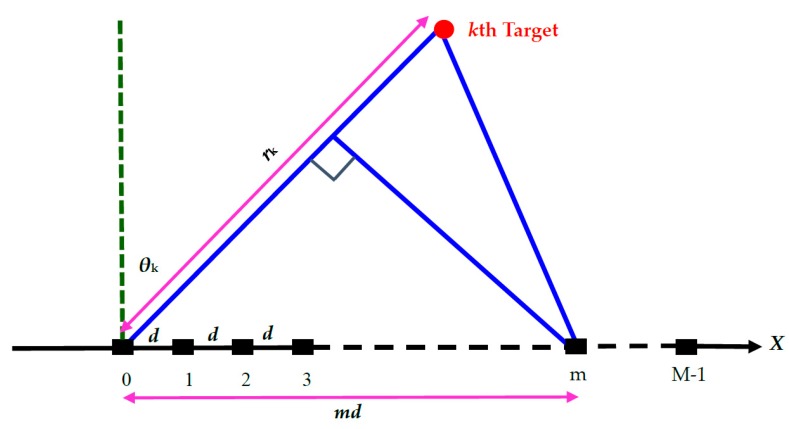
The *k*th near-field target observed by a *M*-element uniform linear array.

Underwater array sensors may be arranged into different patterns such as a straight line, a ring or a plane. This paper considers a uniform linear array (ULA) composed of *M* omnidirectional sensors with the array pitch d, as shown in Figure 1. Suppose that there exists *N* target sources, emitting acoustic signals $A_{k}(t)\exp(j2\pi f_{c}t)$, k=1,⋯,N, with $A_{k}(t)$, exp[•], $f_{c}$ being the amplitude function, exponential function and the carrier frequency, respectively. When these near-field signals are transmitted through sound waves to the ULA, they exhibit different time delays across the sensors. If the sound wave of the *k*th target signal located at a range $r_{k}$ is incident on the array at an angle $\theta_{k}$, where the 0th sensor is used as a reference, then the received signal at the *m*th sensor, sampled at time $t=t_{0}$, is given by [14]

(1) $$y(m)=\sum_{k=1}^{N}A_{k}(t_{0})\exp(j2\pi f_{c}(t_{0}-\frac{\sqrt{{\left(r_{k}-md\sin\theta_{k}\right)}^{2}+{\left(md\cos\theta_{k}\right)}^{2}}}{c}))+z(t_{0})$$

m=0,⋯,M−1, where $j=\sqrt{-1}$, c is the sound speed, $r_{k}$ denotes the distance between the reference sensor and the *k*th target, and $z(t_{0})$ represents the additive white Gaussian noise (AWGN). The objective of this paper is to estimate the bearing $\theta_{k}$ and the range $r_{k}$, k=1,⋯,N, given a single snapshot data y(m), m=0,⋯,M−1, of Equation (1). 

## 3. Proposed Method

The advanced technique for characterizing the time-evolution behavior of nonstationary signals is the Hilbert-Huang transform [18]. It has been applied to estimate a nonstationary signal’s time-frequency parameters, and acquires a great deal of achievements in various fields [19,20,21]. 

### 3.1. Overview of HHT

HHT involves two stages of signal processing: empirical mode decomposition (EMD) and Hilbert spectrum analysis (HSA). EMD is the sifting process that transforms a measured time series into a set of intrinsic mode functions (IMFs). The IMF needs to satisfy two conditions: (1) in the whole dataset, the number of extrema and the number of zero crossings must either equal or differ at most by one; and (2) at any point, the mean value of the envelope defined by the local maxima and the envelope defined by the local minima must be zero. The detailed procedure of EMD includes the following steps [18]:

1. Identify the extrema of the dataset y(t), and form the envelopes defined by the local maxima and minima by the cubic spline interpolation method.
2. Form the mean values $m_{1}(t)$ by averaging the upper envelope and lower envelope, and subtract the mean values from the data to obtain the first component, $h_{1}(t)=y(t)-m_{1}(t)$.
3. Check whether the conditions for an IMF are satisfied. If the first component is not an IMF, let $h_{1}(t)$ be the new data set. Continue with steps 1 and 2 until the first component is an IMF.
4. Let the first IMF component be $c_{1}(t)$. Let $r_{1}(t)=y(t)-c_{1}(t)$. Continue with steps 1–3 until $r_{1}(t)$ is smaller than a predetermined value or becomes a monotonic function from which no more IMF can be extracted. The first component $c_{1}(t)$ contains the finest scale or the shortest period component of the signal. The higher components $c_{2}(t),\cdots ,c_{K}(t)$ contain progressively longer period components. For data with a trend, $r_{K}(t)$ is the trend. At the end of this process, the signal y(t) can be expressed as
  (2) $$y(t)=\sum_{i=1}^{K}c_{i}(t)+r_{K}(t)$$
  where K is the number of IMFs.

HSA is employed to form every single Hilbert spectrum of each IMF to obtain the instantaneous frequency (IF) embedded in the associated IMF. The principle of HSA is described as follows. Considering the real-valued IMF $c_{i}(t)$ for IF extraction, the Hilbert transform is defined as:

(3) $${\hat{c}}_{i}(t)=\frac{1}{\pi }p\int_{-\infty }^{\infty }\frac{c_{i}(\tau )}{t-\tau }d\tau$$

where p is the Cauchy principal value of the singular integral. Combining the real-valued IMF $c_{i}(t)$ with its Hilbert transform ${\hat{c}}_{i}(t)$, an analytic signal is obtained and can be expressed as:

(4) $$z_{i}(t)=c_{i}(t)+j{\hat{c}}_{i}(t)=A_{i}(t)\exp[j\phi_{i}(t)]$$

where $A_{i}(t)$ is the instantaneous amplitude of the analytic signal $z_{i}(t)$, and $\phi_{i}(t)$ is the instantaneous phase. Then the instantaneous frequency (IF) $f_{i}(t)$ is defined as:

(5) $$f_{i}(t)=\frac{1}{2\pi }\frac{d\phi_{i}(t)}{dt}$$

Given that $A_{i}(t)$ and $f_{i}(t)$ are calculated, the original signal y(t) in Equation (2) can be expressed as

(6) $$y(t)=\sum_{i=1}^{K}A_{i}(t)\exp(j\int 2\pi f_{i}(t)dt)$$

Equation (6) enables us to represent both $A_{i}(t)$ and $f_{i}(t)$ as functions of time in three-dimensional plot, in which the amplitude can be contoured on the frequency-time plane, known as Hilbert amplitude spectrum or Hilbert energy spectrum provided that the amplitude is squared.

### 3.2. HHT-Based Target Location Estimation Scheme

In view of our data model given in Equation (1), the received signal is a spatially sampled sequence. Although HHT was originally proposed to analyze time series data, it can be also applied to spatially sampled data. Therefore, the proposed method applies HHT to obtain the spatial instantaneous frequencies from which target location parameters can be estimated. 

In terms of the continuous spatial variable x, Equation (1) can be recast as

(7) $$y(x)=\sum_{k=1}^{N}A_{k}(t_{0})\exp(j2\pi f_{c}(t_{0}-\frac{\sqrt{{\left(r_{k}-x\sin\theta_{k}\right)}^{2}+{\left(x\cos\theta_{k}\right)}^{2}}}{c}))+z(t_{0})$$

After performing EMD, the IMFs $c_{i}(x)$ can be obtained from Equation (2), as:

(8) $$y(x)=\sum_{i=1}^{N}c_{i}(x)+r_{N}(x)$$

It is noted that the total number of IMFs equals the number of target signals, namely K=N. After performing HSA, Equations (3) and (4) become:

(9) $${\hat{c}}_{i}(x)=\frac{1}{\pi }p\int_{-\infty }^{\infty }\frac{c_{i}(\tau )}{x-\tau }d\tau$$

(10) $$z_{i}(x)=c_{i}(x)+j{\hat{c}}_{i}(x)=A_{i}(x)\exp[j\phi_{i}(x)]$$

And Equation (5) becomes the spatial IFs shown below:

(11) $$f_{i}(x)=\frac{1}{2\pi }\frac{d\phi_{i}(x)}{dx}$$

Next, the question “How to extract targets’ parameters from the spatial IFs?” remains. In the followings, we use the time-frequency analysis to exploit the target information via WVD [8,9]. 

Under the noise-free condition, if the distance of the target is much greater than the array aperture ($r_{k}>>Md$), then WVD of the data model in Equation (1) can be derived, in Appendix A, as:

(12) $$WVD_{y}(f,x)=\sum_{k=1}^{N}A_{k}(t_{0})\delta (f-\frac{1}{\lambda }(\sin\theta_{k}-\frac{x\cos^{2}\theta_{k}}{r_{k}}))$$

where λ is the wave length, f is the continuous spatial IF variable, and δ is the Dirac delta function. It should be noted that Equation (12) is only valid if the cross-correlation among the different target’s signal components are neglected, which is not true for WVD. Instead, different IMFs are orthogonal to each other via EMD in HHT [18]. Although the orthogonality property is not theoretically proved, it has been observed in many practical cases and well accepted in the literature [18,19,20,21]. Through the process of EMD, each target’s information is contained in the individual IMF. Therefore, Equation (12) can be viewed as the Hilbert energy spectrum that exhibits total energy concentrated along each target’s spatial IF of

(13) $$f=\frac{1}{\lambda }(\sin\theta_{k}-\frac{x\cos^{2}\theta_{k}}{r_{k}}),\;k=1,\cdots ,N$$

Since the data model in Equation (1) is discrete in space with the sampling rate of $d^{-1}$, the digital spatial frequency $\tilde{f}$ is related to the continuous frequency f by $\tilde{f}=fd$. Therefore, Equation (13) can be recast in terms of $\tilde{f}$ as:

(14) $$\tilde{f}=\frac{1}{\lambda }(d\sin\theta_{k}-\frac{md^{2}\cos^{2}\theta_{k}}{r_{k}}),\;k=1,\cdots ,N$$

Equation (14) is a characteristic function of the chirp signal, which can be used to obtain the intercept $I_{k}$ and slope $S_{k}$ as:

(15) $$I_{k}=\frac{d}{\lambda }\sin\theta_{k}$$

(16) $$S_{k}=-\frac{1}{\lambda }\frac{d^{2}\cos^{2}\theta_{k}}{r_{k}}$$

From Equations (15) and (16), the bearing and range of the *k*th target can be obtained as:

(17) $$\theta_{k}=\sin^{-1}(\frac{\lambda I_{k}}{d})$$

(18) $$r_{k}=\frac{\lambda^{2}I_{k}^{2}-d^{2}}{\lambda S_{k}}$$

In summary, the proposed algorithm can be described as follows:

Step 1. Obtain the IMFs of the snapshot data in Equation (1) by EMD (see Equation (8)).
Step 2. Perform HSA on the IMFs to obtain spatial IFs (see Equations (9)–(11)).
Step 3. Analyze each spatial IF spectrum via linear regression to obtain intercept and slope for the target’s joint bearing and range estimation (see Equations (15)–(18)).

A. Comparison with FFT approach:

Equation (1) represents nonstationary data, because the time delay of the data model varies with sensor location for near-field targets. The proposed method can deal with both far-field and near-field sources. Assumed that the slope of the chirp signal in Equation (13) approaches zero, the spatial IF spectrum shows a stationary and single-frequency signal, and the associated target can be determined as a far-field source. 

However, traditional FFT approach is only suitable for dealing with far-field targets. In this case, sharp peaks of spatial spectrum can be observed to obtain bearing estimate. However, for near-field targets, only flat peaks exhibit, failing to obtain both bearing and range estimates. 

B. Bearing resolution and Range resolution:

Bearing resolution can be derived from Equation (15), under the condition of $r_{k}>>Md$, as

(19) $$R_{\theta }=|\frac{\partial I_{K}}{\partial \theta_{k}}|\approx \frac{d}{\lambda }|\cos\theta_{k}|$$

It can be observed that the angle resolution varies as a function of the target’s bearing. The best bearing resolution is achieved when the target’s angle is from the broadside ($\theta_{k}=0^{{}^{\circ}}$) and the worst bearing resolution occurs when the target’s angle is from the end-of-fire ($\theta_{k}=\pm 90^{{}^{\circ}}$). Moreover, for a fixed angle, bearing resolution improves if the array pitch expressed in terms of wavelength is larger.

Range resolution can be derived from Equation (16) as

(20) $$R_{r}=|\frac{\partial S_{k}}{\partial r_{k}}|=\frac{d^{2}\cos^{2}\theta_{k}}{\lambda r_{k}^{2}}$$

It can be observed that the best range resolution is achieved when target’s angle is from the broadside and the worst range resolution occurs when target’s angle is from the end-of-fire. Moreover, for a fixed angle and array pitch, range resolution is inversely proportional to the square of the target’s range.

C. Array Pitch Constraint

From Equation (14), under the condition of $r_{k}>>Md$, the maximum spatial frequency ${\tilde{f}}_{\max}$ can be approximated as ${\tilde{f}}_{\max}\approx \frac{f_{c}d}{c}$. According to the sampling theory, the space sampling rate $d^{-1}$ must be at least twice the maximum spatial frequency $f_{\max}$, i.e., $d^{-1}\geq 2f_{\max}$. Therefore, the array pitch must be upperly bounded by

(21) $$d\leq \frac{\lambda }{2}$$

Since the resolution and accuracy of the target’s bearing improve as the array aperture increases, and the number of data needs to be sufficiently large as required by HHT, the number of hydrophones is set to be 512. Furthermore, the array pitch should satisfy Equation (21) and the carrier frequency ranges from 40 Hz to 300 kHz for the sound speed of 1482 m/s in underwater applications. Therefore, the array pitch is chosen to be 0.009 cm and the carrier frequency is chosen to be 56 kHz. Table 1 lists the system parameters for computer simulations using MATLAB and for the fabrication of multi-layered ultrasonic sensor chip using ANSYS. 

## 4. Simulation Results

It is assumed that a ULA is used and that the space has two targets: one is from a range of 1000 m and a bearing of 56°, and the other is from a range of 1500 m and a bearing of 10°, respectively, at the signal-to-noise ratio (SNR) of 15 dB. Both the proposed method and the FFT method are performed to compare their performances. 

**Figure 2:**
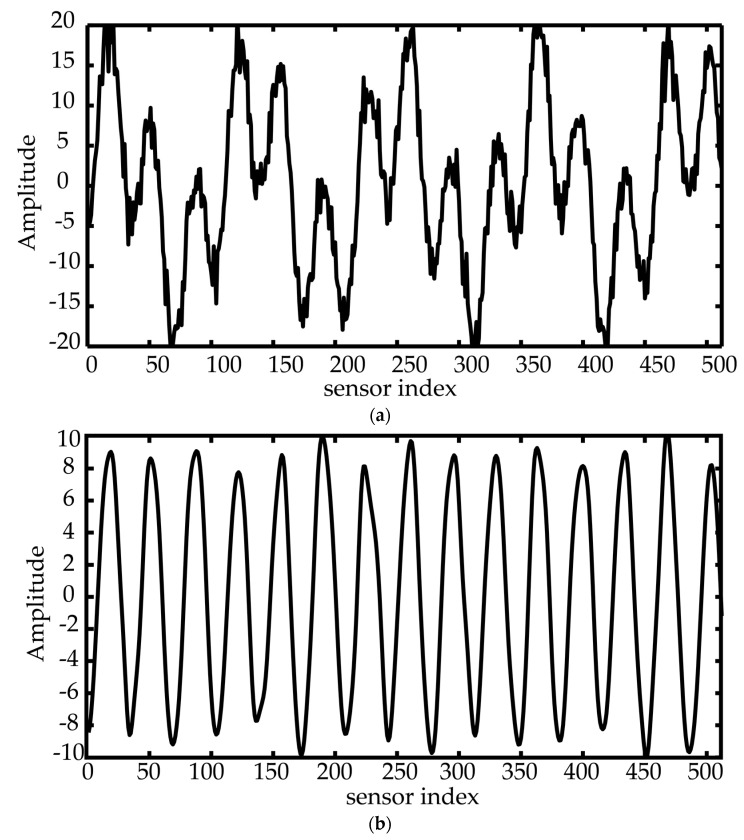

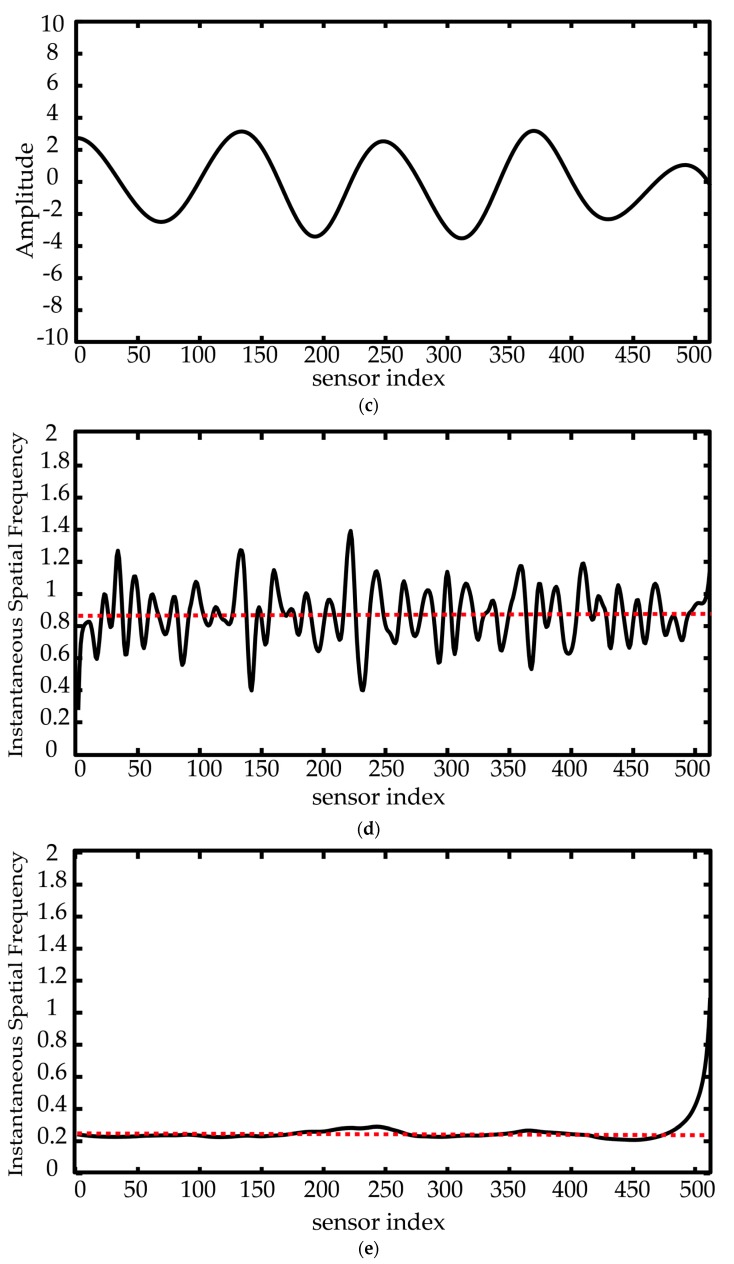
(**a**) The snapshot data; (**b**) The first IMF at the direction angle of 56°; (**c**) The second IMF at the direction angle of 10°; (**d**) Spatial IF (black) with linear regression line (red) at the angle of 56°; (**e**) Spatial IF (black) with linear regression line (red) at the angle of 10°.

Figure 2a shows the snapshot data; Figure 2b shows the first IMF at the direction angle of 56°; Figure 2c shows the second IMF at the direction angle of 10°; Figure 2d shows the continuous spatial IF (measured in the unit of wavelength) at the direction angle of 56° as well as the linear regression line (shown in red). From the linear regression line, the intercept is 0.8217, and the slope is −1.83 × 10^−6^, leading to the estimated angle of 55.9614° and the estimated range of 763.71 m, so the estimation errors are equal to 0.0386° and 236.29 m; and Figure 2e shows the continuous spatial IF (measured in the unit of wavelength) at the direction angle of 10° as well as the linear regression line (shown in red) from which the intercept is 0.17302 and the slope is −3.42 × 10^−6^, leading to an estimated angle of 9.9635° and the estimated range of 1206.53 m, so the estimation errors are equal to 0.0365° and 293.47 m. 

**Figure 3:**
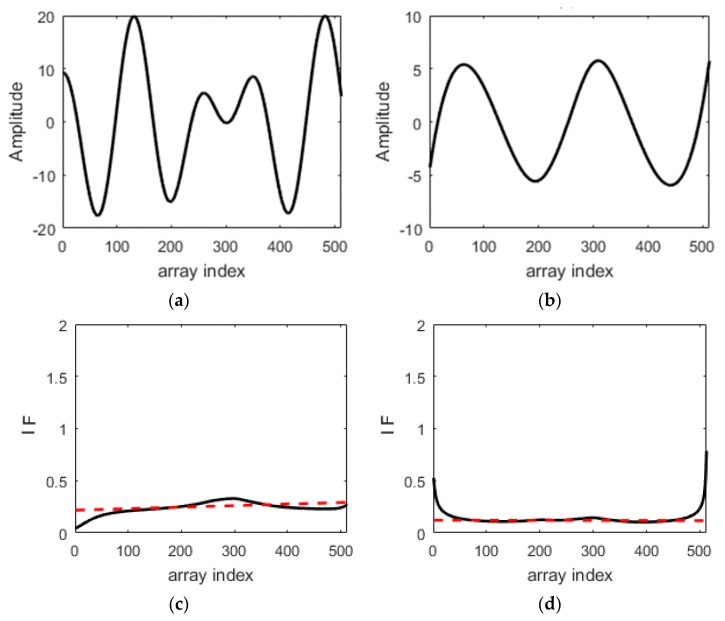
The resulting IMFs (**a**,**b**) as well as spatial IFs (**c**,**d**) using HHT.

Next, two sources, located at the same ranges as those noted in the previous scenario, but at closer directions of 15° and 10°, at SNR of 15 dB, are considered. Figure 3a shows the first IMF at the direction angle of 15°; Figure 3b shows the second IMF at the direction angle of 10°; Figure 3c shows the spatial IF (measured in the unit of wavelength) at the direction angle of 15° as well as the linear regression line (shown in red). From the linear regression line, the intercept is 0.221, leading to the estimated angle of 12.7716°, so the estimation error is 2.284°; and Figure 3d shows the spatial IF (measured in the unit of wavelength) at the direction angle of 10° as well as the linear regression line (shown in red), from which the intercept is 0.1186, leading to an estimated angle of 6.8136°, so the estimation error is 3.186°. It is observed that these two sources with closer directions can be resolved, but the estimation errors are larger. 

**Figure 4:**
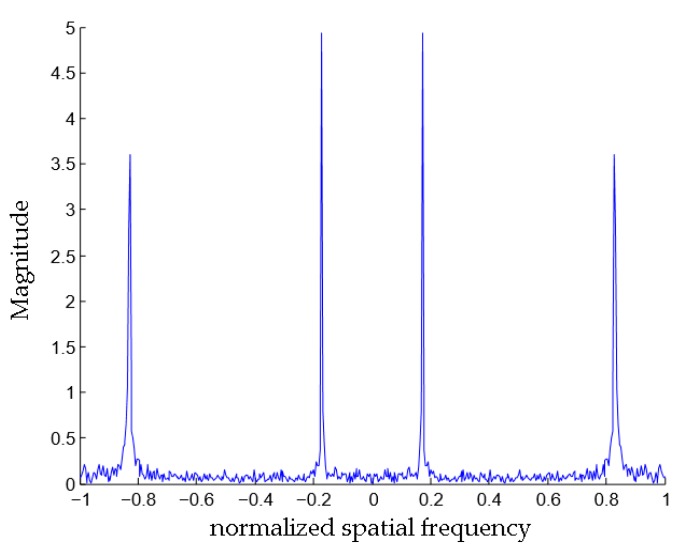
Spatial spectrum obtained from FFT analysis.

Figure 4 shows the result using FFT analysis with the snapshot data shown in Figure 2a. It demonstrates that the range of both targets cannot be estimated. The angle estimate of both targets can be found from the peak locations, from which the angle estimates are 58.21° and 10.66°. As compared to our proposed method, the bearing estimation errors of the FFT analysis are very significant. 

## 5. Experimental Approach

The fabrication of a multi-layered ultrasonic sensor chip is carried out with a 4-inch, single-sided polished bare (111) silicon wafer (supplied by Sino-American silicon product Corporation, Hsinchu Science Park, Taiwan). The wafer was cleaned by RCA (a recipe developed by the RCA Company) and dried with nitrogen gas before beginning the process. A layer of silicon dioxide (SiO_2_) with a thickness 400 nm was first deposited on a Si substrate by RF-magnetron sputtering at room temperature. The Pt (200 nm) bottom electrode was then deposited by RF magnetron sputtering. Subsequently, piezoelectric PZT thin films with a thickness of 5 μm were deposited by using the sol-gel method. The PZT solution (with Zr:Ti ratio = 52:48) was deposited on the SiO_2_-coated substrate by spin-coating at 3000 rpm for 30 s. It was then baked at 350 °C for 60 s on a hot-plate and next crystallized at 650 °C for 120 s for the thin films by using rapid thermal annealing (RTA). Later, a top electrode was formed by sputtering Pt (200 nm)/Ti (10 nm) on the PZT surface. The back-face layer was patterned in a reactive ion etching. Five hundred and twelve hydrophone elements were arrayed in one chip. The chip was packaged on the liquid crystal polymer layer.

**Figure 5:**
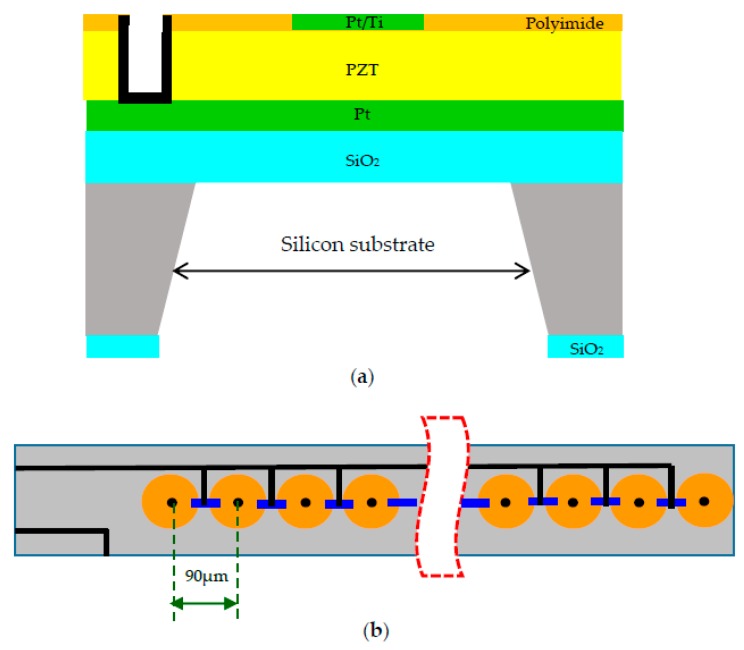
Fabrication process of the hydrophone linear array. (**a**) Schematic cross section illustration of a hydrophone chip. (**b**) Top view of this proposed hydrophone linear array.

Figure 5a shows the schematic structure of a single hydrophone in the acoustic array with MEMS fabrication. The hydrophone frequencies of sensing can be adjusted for a specific application by changing the thickness and width of the diaphragm. The hydrophone is fabricated for a Pt/Ti electrode/PZT/Pt electrode/SiO_2_/Si substrate/SiO_2_ heterojunction structure. The surface of the developed hydrophone is covered with a polyimide to protect against water. These results show that hydrophones of heterojunction structure are promising devices for underwater detection.

Figure 5b shows a schematic diagram of the fabricated diaphragm hydrophone array with 512 linear elements. These elements are adopted through interconnects to allow the individual addressing of each element in a constrained space. The perforation in the two-dimensional (2D) structure is similar to the porosity in the three-dimensional (3D) structure. The transmission and reception characteristics of the hydrophone were investigated using the impulse response in the 0–220 kHz frequency band. It was considered an axial symmetric domain with a diameter of 90 μm and a length of 10 μm. To consider the boundary condition, the edge of a circular plate was fixed. The resonance frequency was evaluated as a function of the cavity diameter. In this work, MEMS-based hydrophone using low-frequency acoustic waves are proposed for underwater application. The hydrophone is fabricated from a PZT diaphragm array with a miniaturized size and optimized length to width ratio. The hydrophone sensing frequencies can be adjusted for different applications by changing the distance between two sensors.

**Figure 6:**
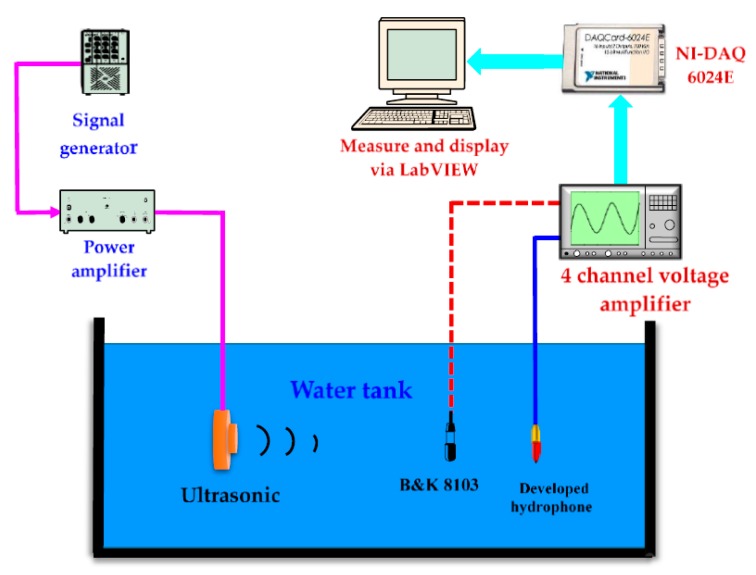
Experimental setup for underwater test of the fabricated hydrophone.

In order to find out the resonant frequency and sensitivity of the fabricated hydrophone, it is immersed into a water tank and connected to a signal generator. The underwater experimental setup [17,22] to measure the characteristics of the fabricated hydrophone is shown in Figure 6. A calibrated hydrophone (B & K 8103) was employed. To measure the frequency response of the hydrophone, the received signals are observed using the Labview system (obtained by acquisition device NI-DAQ 6024E) through four channel amplifiers. The sensitivities of hydrophone are calculated from the generated sound pressure and the output signal. In the present work, we performed FEM (Finite Element Method) using ANSYS to design a hydrophone array, while the properties and dimensions of each hydrophone are used as parameters. For example, at operating frequencies of 40–300 kHz, the maximum cell dimension ranges from 80 to 100 μm. 

**Figure 7:**
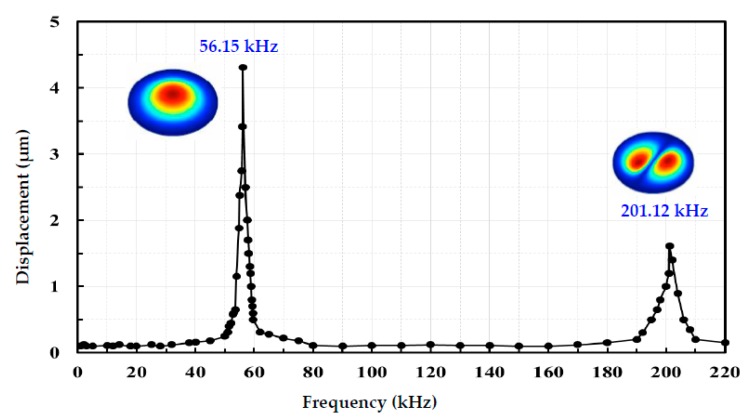
Displacement at sensing membrane in the 1–220 kHz range with a peak-to-peak amplitude output of 200 mV output of a single sensor in the hydrophone linear array.

Figure 7 shows the frequency of the peak-to-peak displacement with a single hydrophone in the ultrasonic array in response to the swept frequencies in the range 0~220 kHz. The transmitter is in a fabricated hydrophone array at a bias of 300 mV and peak-to-peak of 200 mV. The main resonance frequency is located near 56.15 kHz and the second resonance frequency is 201.12 kHz. The experiment results indicate that the displacement increases with frequency (except the resonance frequency). The displacement at the resonance frequency 56.15 kHz is extremely larger than that at any other frequency. A finite element analysis (FEA) model is built by using ANSYS to simulate the resonant frequency of the hydrophone’s active diaphragm. The testing result matches quite well with the simulation, as illustrated in the insert figure of Figure 7. The hydrophone has frequency of 56.05 kHz for 1st resonant mode and frequency of 201.12 kHz for 2nd resonant mode. The optimized diaphragm thickness to width ratio, the 1st and 2nd resonant mode are very close experimentally, which means a nearly ideal transmitting sensitivity is achieved for the hydrophone. Frequency can be adjusted in a specific application by changing the length and width of the sensing diaphragm.

**Figure 8:**
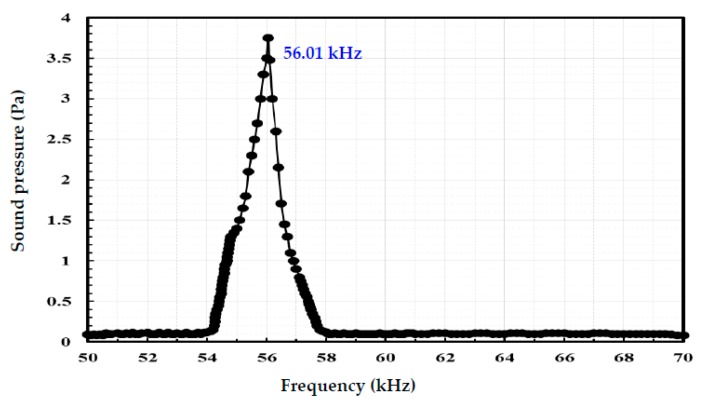
Frequency dependence of output sound pressure of a device in the acoustic array at bias of 300 mV.

Figure 8 shows the measured sound pressure at a single sensor of hydrophone membrane versus frequency. Frequency depends on an output sound pressure of a device in the acoustic array at bias of 300 mV. The hydrophone maximum sound pressure output is 3.75 Pa and the resonance frequency is higher than 56.01 kHz. The two resonance frequencies measured by a vibration displacement test and a sound pressure test are almost the same. Figure 7 and Figure 8 show that the results of the two different measurements are accurate. The response frequency of this electro-acoustical transducer ranges from 1 Hz to 220 kHz, and accordingly this hydrophone can be widely used for underwater frequency and ultrasonic frequency devices.

## 6. Conclusions

A novel approach using HHT is proposed for joint bearing and range estimation of multiple targets based on a uniform linear array of hydrophones. In the past, a MEMS based acoustic linear uniform array of hydrophones for underwater application was proposed by our research team [17,23]. The micro hydrophone array has a very compact size, suitable for AUV operations. In this paper, the system parameters are considered to fit ULA data model in underwater application as well as a MEMS design technique. This proposed target location estimation method has the following advantages: (1) only a single snapshot of data is needed; and (2) real-time processing is feasible. The proposed algorithm achieves the transformation of a very complicated nonlinear estimation problem to a simple nearly linear one via TFD theory, and verifies the results with HHT. Theoretical discussions of resolution issue in this paper can facilitate the design of a MEMS sensor with high sensitivity.

## Figures and Tables

**Table 1:** List of System Parameters.

Parameters	Unit	Value
sound carrier frequency, $f_{c}$	kHz	56
sound speed, c	m/s	1482
sound wavelength, λ	cm	2.65
array pitch, *d*	cm	0.009
array element number, *M*	count	512
array aperture , *Md*	cm	4.6

## Appendix A. Derivation of Equation (12)

The time delay of the *k*th target from Equation (7) can be written as:

(A1) $$\tau_{k}(x)=\frac{\sqrt{{\left(r_{k}-x\sin\theta_{k}\right)}^{2}+{\left(x\cos\theta_{k}\right)}^{2}}}{c}$$

Under the condition of $r_{k}>>Md\geq x$, we can use Taylor series expansion to write Equation (A1) as (saving terms up to the first order):

(A2) $$\begin{array}{l} \tau_{k}(x)=\frac{1}{c}(r_{k}-x\sin\theta_{k}){\left(1+\frac{{\left(x\cos\theta_{k}\right)}^{2}}{{\left(r_{k}-x\sin\theta_{k}\right)}^{2}}\right)}^{1/2}\\ \approx \frac{1}{c}(r_{k}-x\sin\theta_{k})[1+\frac{{\left(x\cos\theta_{k}\right)}^{2}}{2{\left(r_{k}-x\sin\theta_{k}\right)}^{2}}]\\ =\frac{1}{c}[r_{k}-x\sin\theta_{k}+\frac{{\left(x\cos\theta_{k}\right)}^{2}}{2(r_{k}-x\sin\theta_{k})}]\\ \approx \frac{1}{c}[r_{k}-x\sin\theta_{k}+\frac{{\left(x\cos\theta_{k}\right)}^{2}}{2r_{k}}] \end{array}$$

In a noise-free case, by substituting Equation (A2) into Equation (7), we can obtain:

(A3) $$y(x)=\sum_{k=1}^{N}A_{k}(t_{0})\exp(j2\pi f_{c}(t_{0}-\frac{1}{c}(r_{k}-x\sin\theta_{k}+\frac{{\left(x\cos\theta_{k}\right)}^{2}}{2r_{k}})))$$

The data in Equation (A3) represents a sum of *N* chirp signals, each with spatial IF of $\frac{f_{c}}{c}(\sin\theta_{k}-\frac{x\cos^{2}\theta_{k}}{r_{k}})$. In time-frequency analysis [8,9], for a single chirp signal, WVD is a Dirac delta function with total energy concentrated along its IF. As for a sum of multiple chirp signals, its WVD is the sum of self and cross terms. Each self term is a delta function with energy concentrated along its associated IF. Cross terms represent energy spread due to the cross-correlation of different signal components, which needs to be reduced or removed. At the ideal case of these cross terms being absent, WVD of y(x) becomes [8,9]

(A4) $$WVD_{y}(f,x)\approx \sum_{k=1}^{N}A_{k}(t_{0})\delta (f-\frac{f_{c}}{c}(\sin\theta_{k}-\frac{x\cos^{2}\theta_{k}}{r_{k}}))$$
